# The Self-Face Paradigm Improves the Performance of the P300-Speller System

**DOI:** 10.3389/fncom.2019.00093

**Published:** 2020-01-15

**Authors:** Zhaohua Lu, Qi Li, Ning Gao, Jingjing Yang

**Affiliations:** School of Computer Science and Technology, Changchun University of Science and Technology, Changchun, China

**Keywords:** brain-computer interface (BCI), event-related potential, famous face, P300-speller, self-face

## Abstract

**Objective:** Previous studies have shown that the performance of the famous face P300-speller was better than that of the classical row/column flashing P300-speller. Furthermore, in some studies, the brain was more active when responding to one's own face than to a famous face, and a self-face stimulus elicited larger amplitude event-related potentials (ERPs) than did a famous face. Thus, we aimed to study the role of the self-face paradigm on further improving the performance of the P300-speller system with the famous face P300-speller paradigm as the control paradigm.

**Methods:** We designed two facial P300-speller paradigms based on the self-face and a famous face (Ming Yao, a sports star; the famous face spelling paradigm) with a neutral expression.

**Results:** ERP amplitudes were significantly greater in the self-face than in the famous face spelling paradigm at the parietal area from 340 to 480 ms (P300), from 480 to 600 ms (P600f), and at the fronto-central area from 700 to 800 ms. Offline and online classification results showed that the self-face spelling paradigm accuracies were significantly higher than those of the famous face spelling paradigm at superposing first two times (*P* < 0.05). Similar results were found for information transfer rates (*P* < 0.05).

**Conclusions:** The self-face spelling paradigm significantly improved the performance of the P300-speller system. This has significant practical applications for brain-computer interfaces (BCIs) and could avoid infringement issues caused by using images of other people's faces.

## Introduction

A brain-computer interface (BCI) is a communication technology based on brain activity. BCIs allow severely disabled patients, especially patients with amyotrophic lateral sclerosis, to send messages or control external devices without physical actions (Thompson et al., 2013; Rosenfeld and Wong, 2017; Lazarou et al., 2018). BCIs can also help restore function in patients with severe motor disabilities, including patients with spinal cord injury, stroke, neuromuscular disorder, and limb amputation (Takeuchi et al., 2015; Carelli et al., 2017; Wang et al., 2019). In recent years, some studies have used BCIs for enhancing clinical communication assessments in patients with disorders of consciousness (Wang et al., 2017; Jeunet et al., 2018). BCIs are commonly based on electroencephalogram (EEG) that is recorded non-invasively via electrodes placed on the surface of the head (Waldert, 2016).

The P300 event-related potential (ERP) induced by an oddball paradigm is commonly used in non-invasive BCI systems (Bernat et al., 2001). Farwell and Donchin (1988) first applied the P300 potential to a BCI system; they achieved a character-spelling system based on the P300, which was called the P300-speller system. The users attend to a cell of the matrix (that is, a target character) and count the number of times it is intensified. In this system, the probability of the intensified row/column containing the target character is 1/6 (a matrix of 6 rows and 6 columns), which is an oddball event, which therefore would induce P300 potentials; the system can then output a character by analyzing the P300 potentials. However, the system was not satisfactory due to its low speed and variable accuracy.

A number of studies have attempted to design different paradigms to improve the performance of the P300-speller system (Allison and Pineda, 2003, 2006; Sellers et al., 2006; Salvaris and Sepulveda, 2009; Li et al., 2019). Kaufmann et al. (2011) introduced the famous face paradigm into the P300-speller system and found that its performance was markedly superior to that of the conventional P300-speller system, because the face stimulus also induced other ERPs (e.g., the N170) in addition to an increased P300 amplitude, which enhanced the waveform difference between the target and non-target characters. Subsequently, Jin et al. (2012) compared the performance of P300-speller system between the stimulus types involving a famous face, character flashing, and character movement, and the results showed that the system performed significantly better under the famous face condition than under the other two conditions. Recently, Speier et al. (2017) compared the stimulus types in an online classification of the P300-speller, and the results showed that famous faces stimuli yielded superior results than that with both standard and character inversion stimuli. Some researchers have attempted to optimize the face paradigm to improve the performance of the P300-speller system. For example, Jin et al. (2014b) designed a new stimulus presentation based on facial expression changes, to reduce adjacent interference annoyance and fatigue. Li et al. (2015) combined chromatic properties and the famous face spelling paradigm, which improved the performance of the P300-speller system.

Studies on human face recognition have shown that the brain has specialized cognitive processing for one's own face as compared with other faces. When participants searched for their own face vs. another face, they consistently processed their own face faster than other faces (Tong and Nakayama, 1999). Prior fMRI studies have shown that neural activity was enhanced over the frontal central area for self-face recognition as compared to other face recognition (Kircher et al., 2001). Some ERP studies on human face recognition have shown that the self-face induced greater ERP amplitudes than did other faces. The P300 is more sensitive to the self-face than to other faces (Ninomiya et al., 1998). For example, several studies have found that one's own face elicits a larger P300 amplitude than does a famous face (Caharel et al., 2005; Sui et al., 2006; Miyakoshi et al., 2008; Keyes et al., 2010; Tacikowski et al., 2011). The N170 is face-specific component that reflects facial perception (Bentin and Deouell, 2000; Schweinberger et al., 2002; Herzmann et al., 2004; Carbon et al., 2005). In Caharel et al.'s (2005) study on face processing, the self-face induced a larger N170 amplitude than did famous and unknown faces, distinguishing the self-face from famous and unknown faces. Other studies have also found that the self-face induced a larger N170 amplitude than did other faces (Miyakoshi et al., 2008; Keyes et al., 2010).

Thus, existing studies of face recognition have suggested that the brain is more active in response to the self-face than to a famous face. In the present study, we designed a new spelling paradigm based on self-face stimuli, in which we replaced the famous face with the self-face, to investigate whether the use of the self-face could improve the performance of the P300-speller system. The control paradigm was that of the famous face spelling paradigm. We analyzed the ERP waveforms induced in the self-face and famous face spelling paradigms and compared the classification accuracies between the two spelling paradigms.

## Materials and Methods

### Subjects

A total of 20 subjects participated in the study; of these, one group (*n* = 10, three men, aged 20–28 years, mean 24.4 years) participated in the offline experiment, and the other group (*n* = 10, six men, aged 22–29 years, mean 25.6 years) participated in the online experiment. The subjects did not have any known neurological disorders and had a normal or corrected-to-normal vision. This study was carried out in accordance with the recommendations of the ethics committee of Changchun University of Science and Technology, which approved the protocol. All subjects gave written informed consent in accordance with the Declaration of Helsinki. All subjects were native Chinese speakers but were familiar with the Western characters used in the display.

### Spelling Paradigms

**Figure 1:**
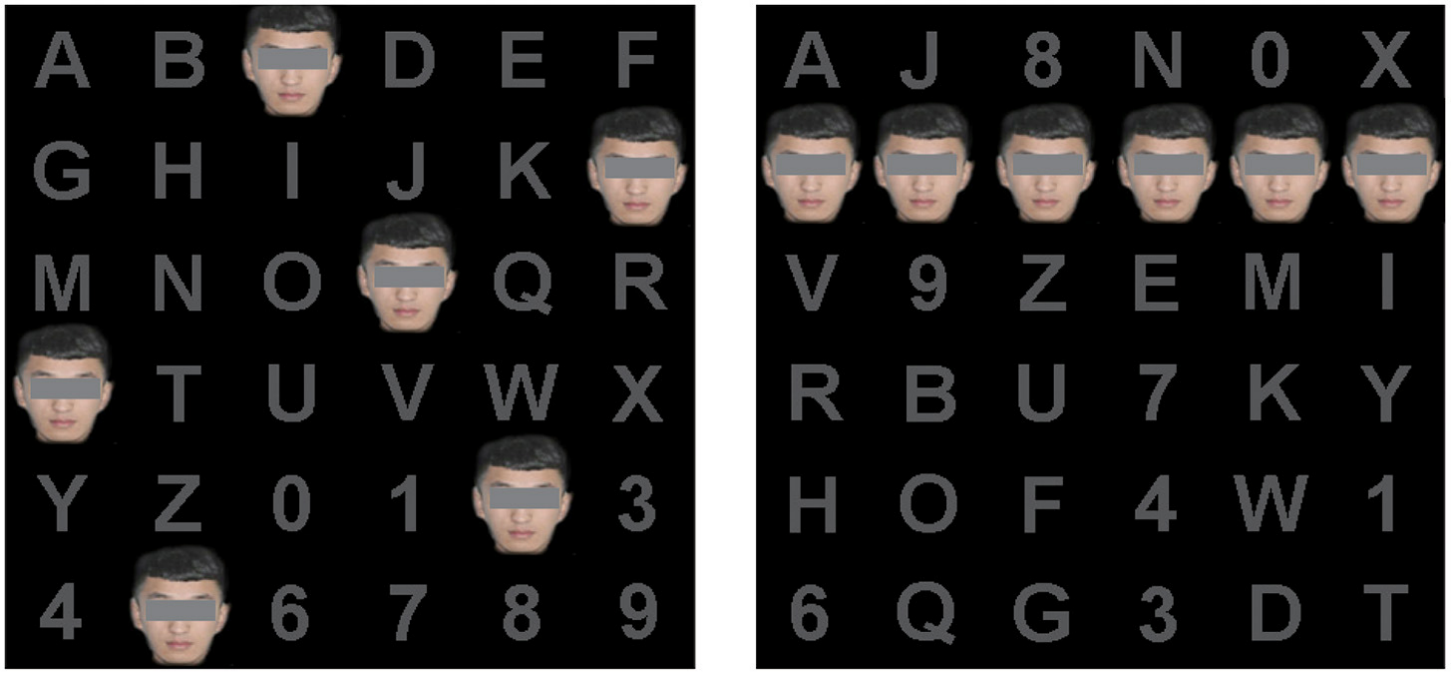
The spelling paradigm. The left figure is the actual spelling matrix, and the right figure is a virtual matrix of the spelling paradigm. The figure shows the self-face paradigm in which the facial photograph is that of a subject.

We designed two P300-speller paradigms based on the conventional P300-speller paradigm. For each paradigm, 36 characters were presented in a 6 × 6 matrix subtended at a 13.4° × 19.4° (24 × 1.5 cm) visual angle on a 19-in screen with a refresh rate of 60 Hz (Figure 1). In the first paradigm, the rows or columns of the characters were covered with pictures of the subject's self-face while they were intensified (self-face spelling paradigm, as shown in Figure 1; the subject has provided permission to publish his facial photograph in Figure 1). In the control spelling paradigm (the famous face spelling paradigm), the characters were covered with the famous face, and the paradigm's setup was the same as that of the self-face spelling paradigm.

We chose a picture of Ming Yao, a sports star, as the famous face. The subjects' self-face was photographed with a digital camera for the self-face paradigm. All facial images were frontal and showed a neutral expression. These photographs were processed to remove the background and everything below the neck in Adobe Photoshop (Adobe Systems, Inc. San Jose, CA, USA).

In our study, the characters were intensified according to the rows and columns of a virtual matrix (Figure 1, right). In the virtual matrix, the characters were randomly rearranged into a new matrix in which the characters of the same row or the same column in the traditional matrix were positioned as far away as possible. Therefore, the rows or columns were six random characters in the actual matrix (Figure 1, left), which mitigates the problem of adjacency flashing (Townsend et al., 2010). The rows and columns of the virtual matrix flashed consecutively in a pseudo-random order. The stimulus onset asynchrony of each paradigm was set to 250 ms, in which each character was covered with a picture of a face for 200 ms and then reverted to a gray character for 50 ms.

### Procedure

Each subject sat in a comfortable chair, ~70 cm from the front of the computer monitor, in a shielded room. During data acquisition, subjects were asked to relax and avoid unnecessary movement. The subjects' task was to focus on the target character and silently count the number of times the target characters were covered with faces during stimulus presentation.

**Figure 2:**
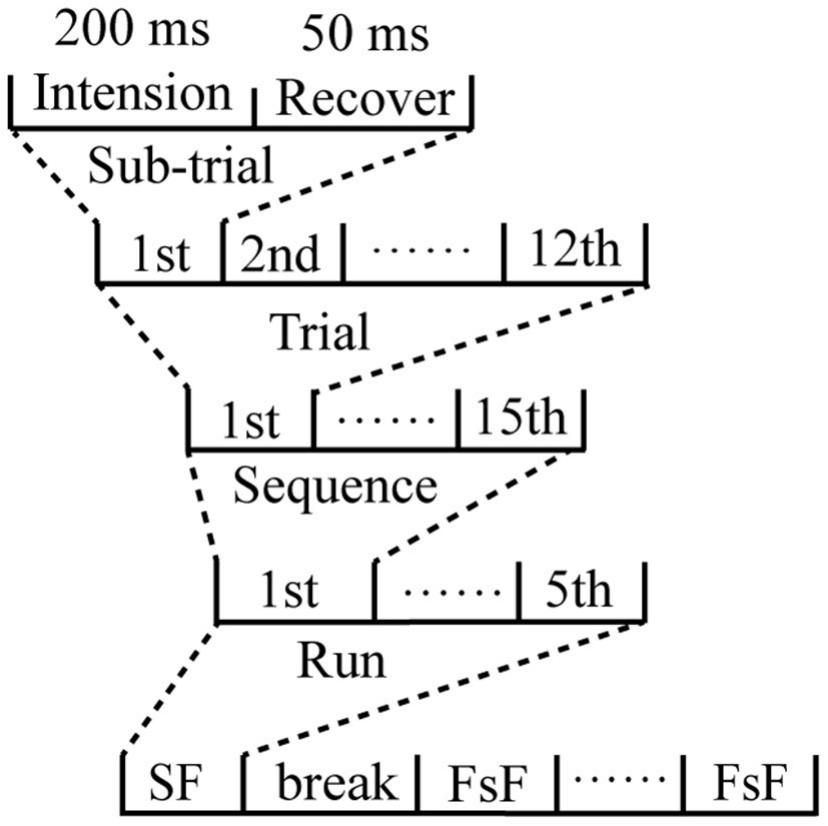
Diagrammatic representation of the time-course of the experiment.

In the offline experiment, one flash of a row or column was referred to as a sub-trial. The flash of a row or column that included the target character was defined as a target sub-trial, and the flash of a row or column without the target character was defined as a non-target sub-trial. Six rows and six columns flashed once (12 flashes) as a trial, and the trial was repeated 15 times as a sequence. Thus, each sequence consisted of 180 flashes of rows or columns to output a target character. During the experiment, each spelling paradigm was conducted four times, and each time, a five-character word was spelled out, which was considered a run (Figure 2). The runs of the two paradigms were counted alternately to control for potential habituation effects. Participants were allowed to take a 5-min break between runs.

In the online experiment, each subject completed training and testing phases for the famous face and self-face spelling paradigms. In the training phase, there were four runs, and each run contained 20 sequences (whereby one character was revealed per sequence); that is, there were 20 characters in a run and a total of 80 characters in the training phase for each spelling paradigm, which were used to obtain the classifier. The test phase output a total of 30 characters by the trained classifier. In addition, trials were only repeated twice in each sequence for both the training and testing phases.

### Data Acquisition

**Figure 3:**
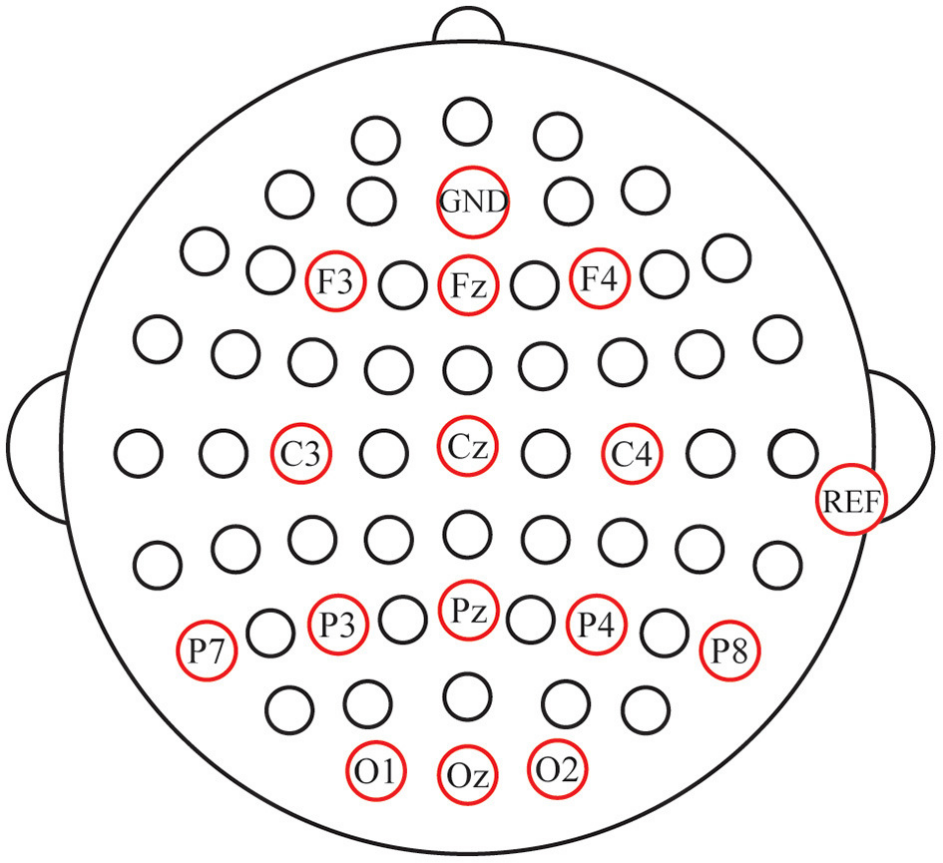
Configuration of electrode positions.

EEG signals were recorded with a NeuroScan amplifier (SynAmps 2, NeuroScan Inc., and Abbotsford, Australia). All signals were digitized at a rate of 250 Hz, and band-pass filtered between 0.1 and 100 Hz. Fourteen-channel (Fz, F3, F4, C3, Cz, C4, P7, P8, P3, P4, Pz, O1, Oz, and O2, Figure 3) EEG data were recorded with the AFz as the ground and the right mastoid as the reference electrode position. Horizontal eye movements were measured by deriving the electrooculogram (EOG) from a pair of horizontal EOG (HEOG) electrodes placed at the outer canthi of both the left and right eyes. Vertical eye movements and eye blinks were detected by deriving an EOG signal from a pair of vertical EOG (VEOG) electrodes placed ~1 cm above and below the subject's left eye. The impedance was maintained below 5 KΩ.

### Feature Extraction Procedure

For offline data, the classification performance of the speller depends not only on the amplitude of ERPs elicited by the target stimulus but also on the difference in ERP amplitudes elicited by the target and non-target stimuli. Thus, the analysis of *R*^2^ values can provide the mathematic foundation for selecting channels and the features of each channel. The r-squared is calculated by formula (1)

(1) $$\begin{array}{r} r^{2}={\left(\frac{\sqrt{N_{1}N_{2}}}{N_{1}+N_{2}}\times \frac{mean\left(x_{1}\right)-mean\left(x_{2}\right)}{std\left(x_{1}⋃x_{2}\right)}\right)}^{2} \end{array}$$

where *N*_1_ and *N*_2_ represent the sample size of the target and non-target stimuli, respectively; *x*_1_and *x*_2_ are features vector of the target and non-target stimuli, respectively.

According to the results of the r-squared values, ERP data of different time windows were down-sampled from 250 to 62.5 Hz by selecting every four samples, and the feature vector was *N*_*p*_ × *N*_*c*_, where *N*_*p*_ represents the sample points within the selected time window, and *N*_*c*_ represents the number of channels. For online data, the EEG data were first filtered between 0.1 and 30 Hz using a third-order Butterworth bandpass filter, then down-sampled from 250 to 50 Hz. We extracted the EEG data from 200 to 800 ms after stimuli onset as the vector feature.

### Classification Scheme

Bayesian linear discriminant analysis (BLDA) was used to classify the EEG data in the experiment. BLDA is an extension of Fisher's linear discriminant analysis that avoids over-fitting. The details of the algorithm have been described elsewhere (Hoffmann et al., 2008; Jin et al., 2014a). We used 4-fold cross-validation to calculate the individual accuracy in the offline experiment.

### Information Transfer Rate

Information transfer rate (ITR) is generally used to evaluate the communication performance of a BCI system and is a standard measure that accounts for accuracy, the number of possible selections, and the time required to make each selection (Thompson et al., 2013). The ITR (bits min^−1^) can be calculated as follows:

(2) $$\begin{array}{r} ITR=\frac{60\left(P\;\log_{2}\left(P\right)+\left(1-P\right)\;\log_{2}\frac{1-P}{N-1}+\log_{2}N\right)}{T} \end{array}$$

where *P* denotes the probability of recognizing a character, *T* is the time taken to recognize a character, and *N* is the number of classes (*N* = 36).

### Data Analysis

A one-way repeated measure ANOVA with the within-subjects two factors of spelling paradigm (self-face and famous face spelling paradigms) and electrodes (electrodes were based on the waveform of ERPs elicited by target stimuli) was used to compare the difference in ERP amplitudes between self-face and famous face spelling paradigms acquired by subtracting the waveforms elicited by non-target stimuli from that by target stimuli. The comparison of classification accuracy and ITR in offline and online experiments was conducted by a paired *T*-test. The statistical analyses were conducted using the SPSS version 19.0 software package (SPSS Inc., Chicago, IL, USA).

## Results

### ERP Results

**Figure 4:**
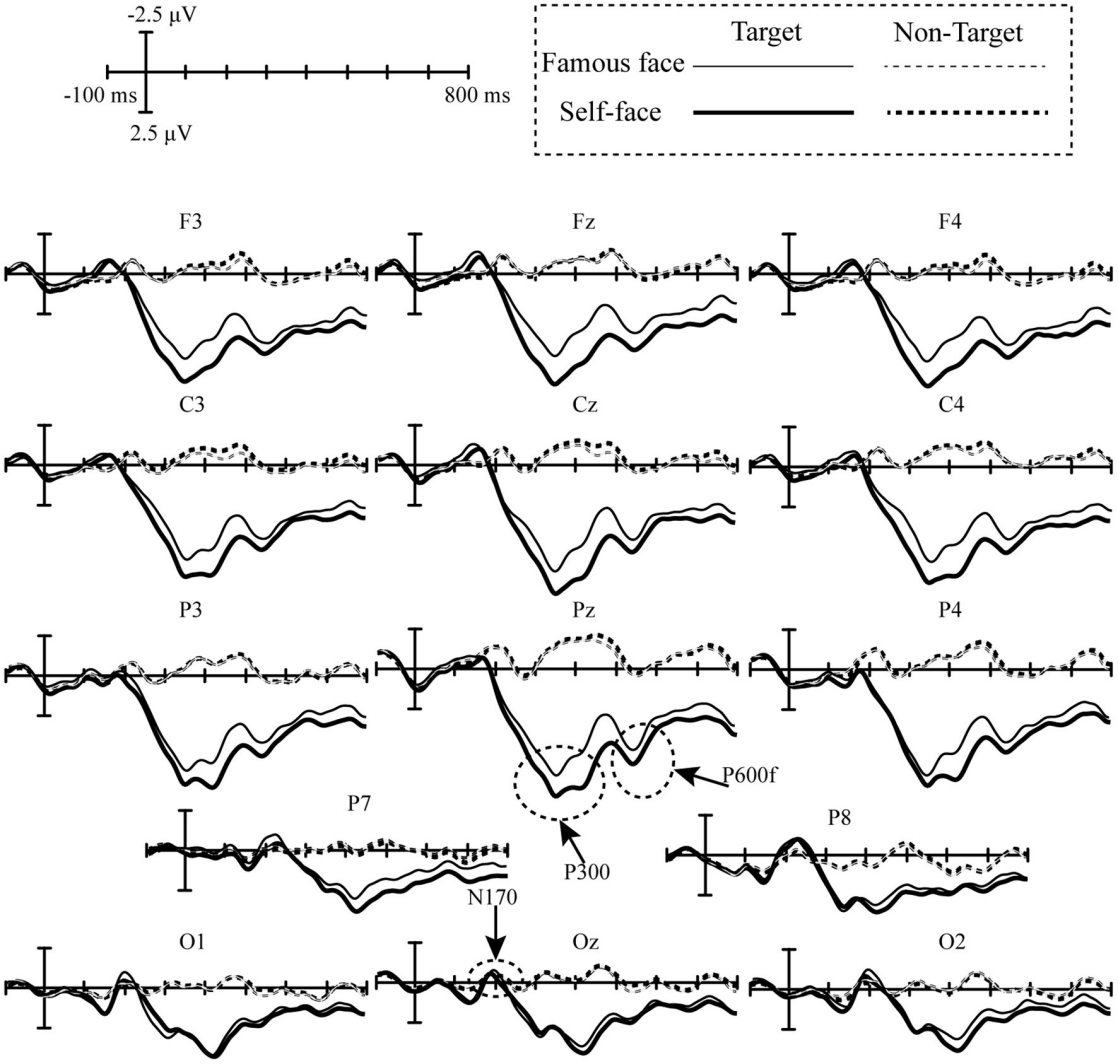
Superimposed grand-averaged event-related potentials elicited by the target and non-target stimuli over 14 electrodes in the self-face and famous face spelling paradigms.

Figure 4 displays the superimposed grand-averaged waveform elicited by target and non-target stimuli in the self-face and famous face spelling paradigms. A clear negative peak was observed at O1, Oz, and Oz between 150 and 200 ms, which is indicative of the N170 potential. In addition, we observed a clear positive peak at all electrodes between 200 and 500 ms, which is indicative of the P300 potential, and the other positive peak was observed between 500 and 600 ms, at F3, Fz, F4, C3, Cz, C4, P3, Pz, and P4, which is similar to the P600f potential.

**Figure 5:**
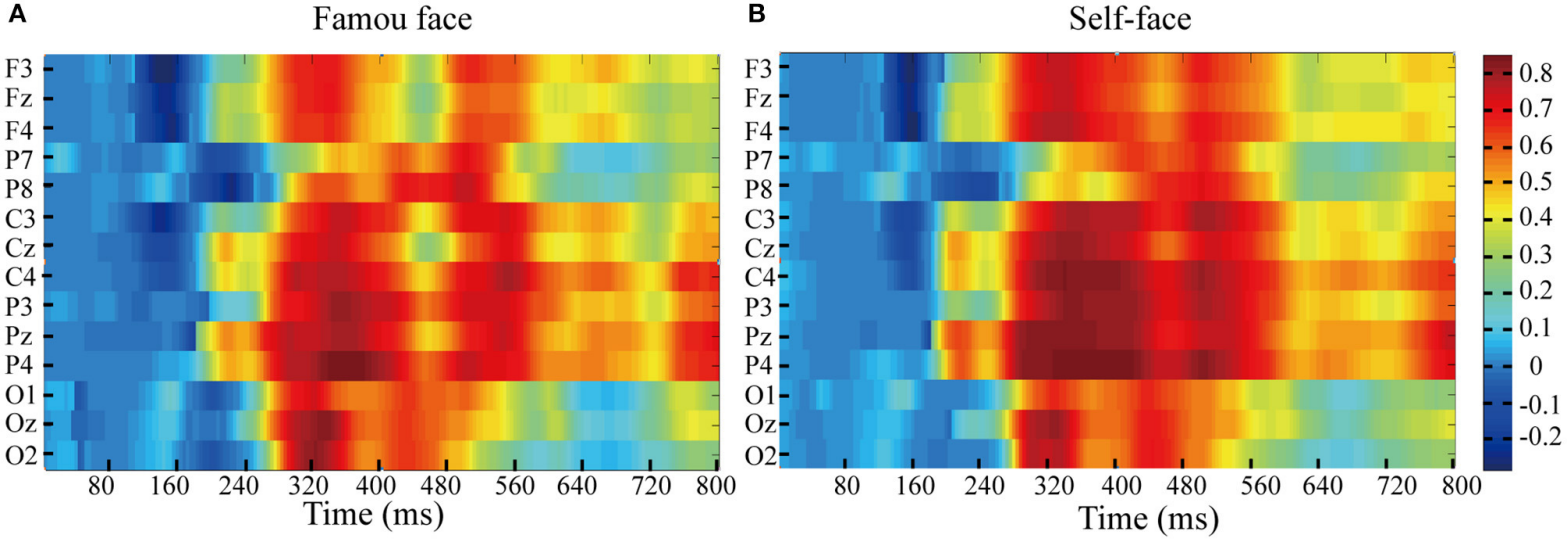
*R*^2^ values of ERPs in response to the target and non-target stimuli between 0 and 800 ms from EEG data of all subjects in the famous face and self-face spelling paradigms. **(A)** *R*^2^ values of ERPs for the famous face spelling paradigm. **(B)** *R*^2^ values of ERPs for the self-face spelling paradigm.

Feature differences in the ERPs elicited by target and non-target stimuli in the famous face and self-face spelling paradigms were indicated by the r-squared values (Figure 5). As seen in Figure 5, we observed that the feature differences in the ERPs elicited by target and no-target stimuli were mainly between 200 and 800 ms at all electrodes for both the famous face and self-face spelling paradigms. To represent the positive and negative deflections of ERP amplitude and to allow for richer visual information, we set the *R*^2^ value corresponding to the negative ERP amplitude value as a negative value.

**Figure 6:**
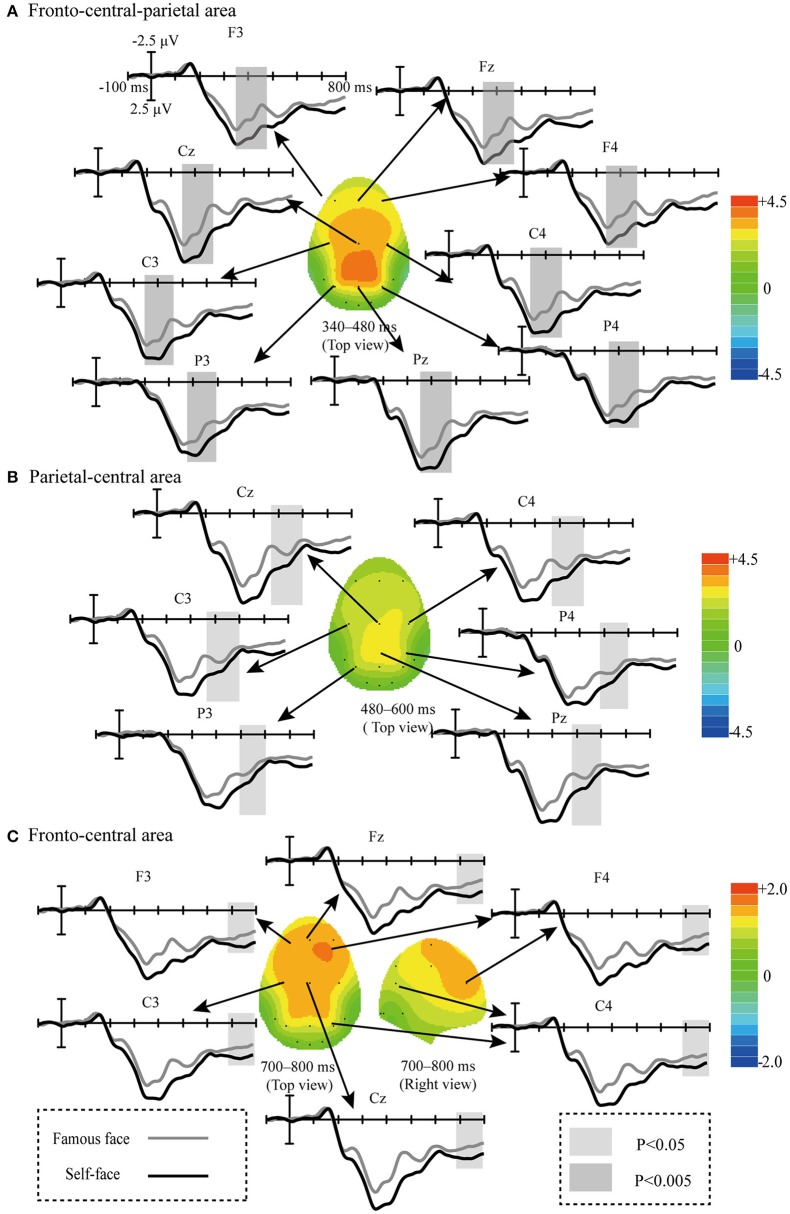
Comparison of waveforms (ERP_Target_ – ERP_Non−target_) elicited by the target and non-target stimuli in the self-face and famous face spelling paradigms, and scalp topographies from difference waveforms. Difference waveforms were calculated by subtracting the ERPs of the famous face spelling paradigm from those of the self-face spelling paradigm. **(A)** The fronto-central-parietal area at 340–480 ms. **(B)** The parietal-central area at 480–600 ms. **(C)** The fronto-central area at 700–800 ms.

Figure 6 displays the scalp topographic regions that corresponded to significant differences between the waveforms elicited in the self-face and famous face spelling paradigms. Significant differences were observed in three regions corresponding to three time periods after stimulus presentation, as follows: the fronto-central-parietal area from 340 to 480 ms [*F*_(1, 9)_ = 14.54, *P* < 0.005; Figure 6A]; the parietal-central area from 480 to 600 ms [F_(1, 9)_ = 8.018, *P* < 0.05; Figure 6B]; and the fronto-central area from 700 to 800 ms [F_(1, 9)_ = 6.023, *P* < 0.05; Figure 6C].

### Classification Results

**Figure 7:**
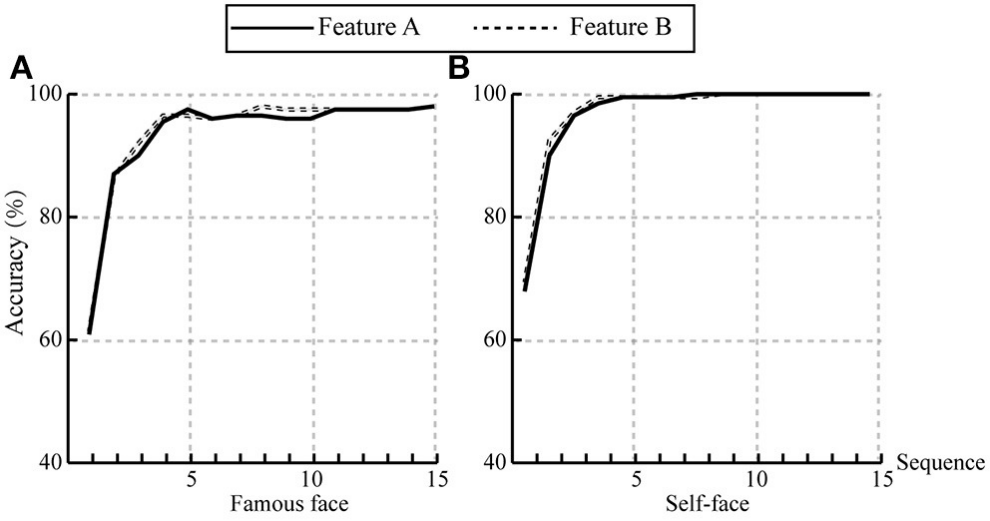
The comparison of classification accuracies based on feature vector A and feature vector B. **(A)** The average accuracies across all subjects in the famous face spelling paradigm. **(B)** The average accuracies across all subjects in the self-face spelling paradigm.

Based on the results of the r-squared values, we compared the classification accuracies based on two feature vectors, as follows: the feature vector A was 25 × 12 (time window of 200–700 ms, channels F3, Fz, F4, C3, Cz, C4, P3, Pz, P4, O1, Oz, and O4); the feature vector B was 45 × 14 (time window of 0–800 ms, 14 channels). The results of classification accuracies based on feature A and feature B are shown in Figure 7, which shows the average accuracies across all subjects at each sequence in famous face and self-face spelling paradigms. There was no significant difference in accuracy between feature A and feature B in the two spelling paradigms.

**Figure 8:**
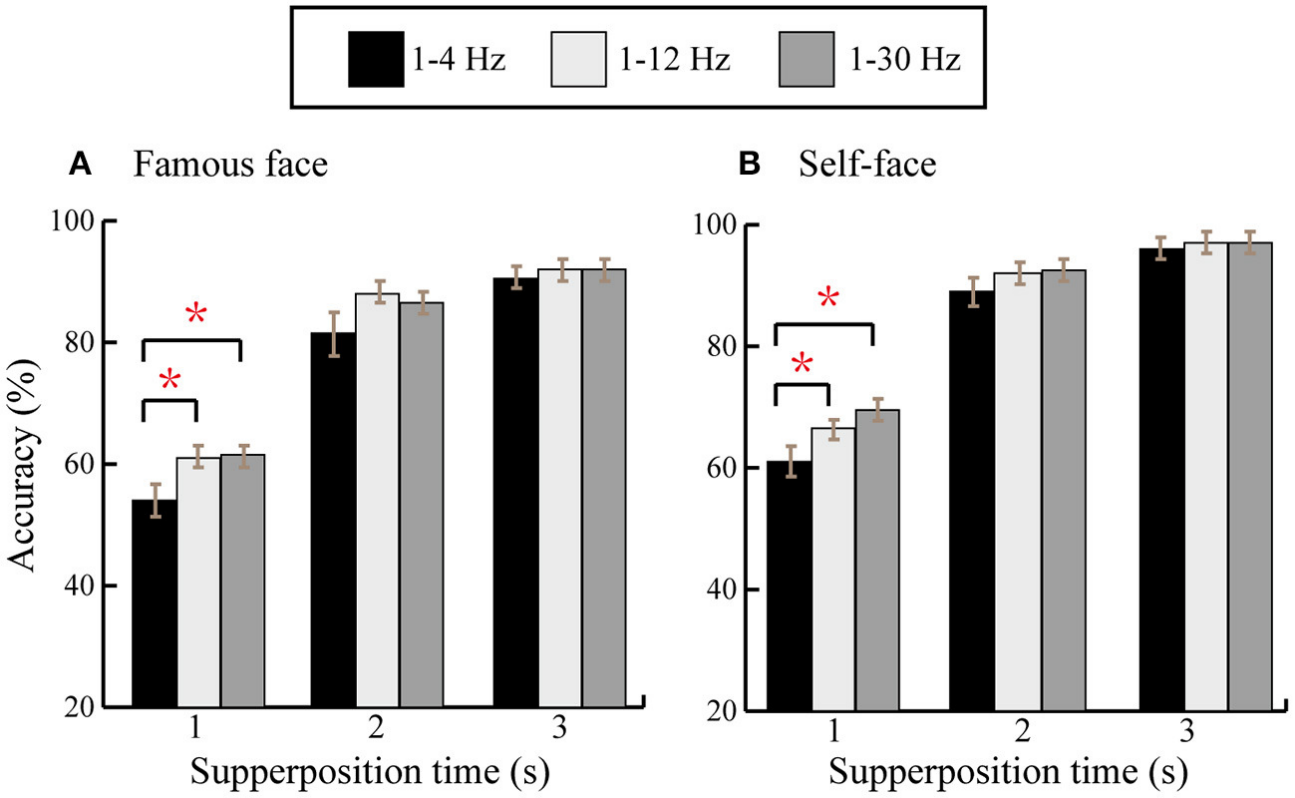
Average offline classification accuracies across all subjects at the first three superpositions for 1–4, 1–12, and 1–30 Hz. **(A)** The comparison of accuracies between three frequency band filters in the famous face spelling paradigm. **(B)** The comparison of accuracies between three frequency band filters in the self-face spelling paradigm. ^*^A significant difference in accuracy between two frequency bands.

Previous work has shown that the frequency band for the P300 is mainly between 1 and 10 Hz (Basar-Eroglu et al., 1992) and different band passes have been used to filter EEG data to acquire better classification accuracy, such as 1–4, 1–12, and 1–30 Hz (Jin et al., 2017). In this study, we compared the classification accuracies at the first three superpositions (superposition times represent the number of trials, that is, the repeating times of 6 rows/columns flashing) between 1–4, 1–12, and 1–30 Hz for the famous face and self-face spelling paradigms (Figure 8). We found that the average accuracy at 1–12 Hz was larger than that at 1–4 Hz, and the average accuracy at 1–30 Hz was larger than that at 1–4/1–12 Hz for the first three superpositions in the two spelling paradigms except for the accuracies between 1–12 and 1–30 Hz at two superpositions in the famous face spelling paradigm. The paired *t*-test results revealed a significant difference for classification accuracy between 1–4 and 1–12 Hz/1–30 Hz in the famous face and self-face spelling paradigms.

**Figure 9:**
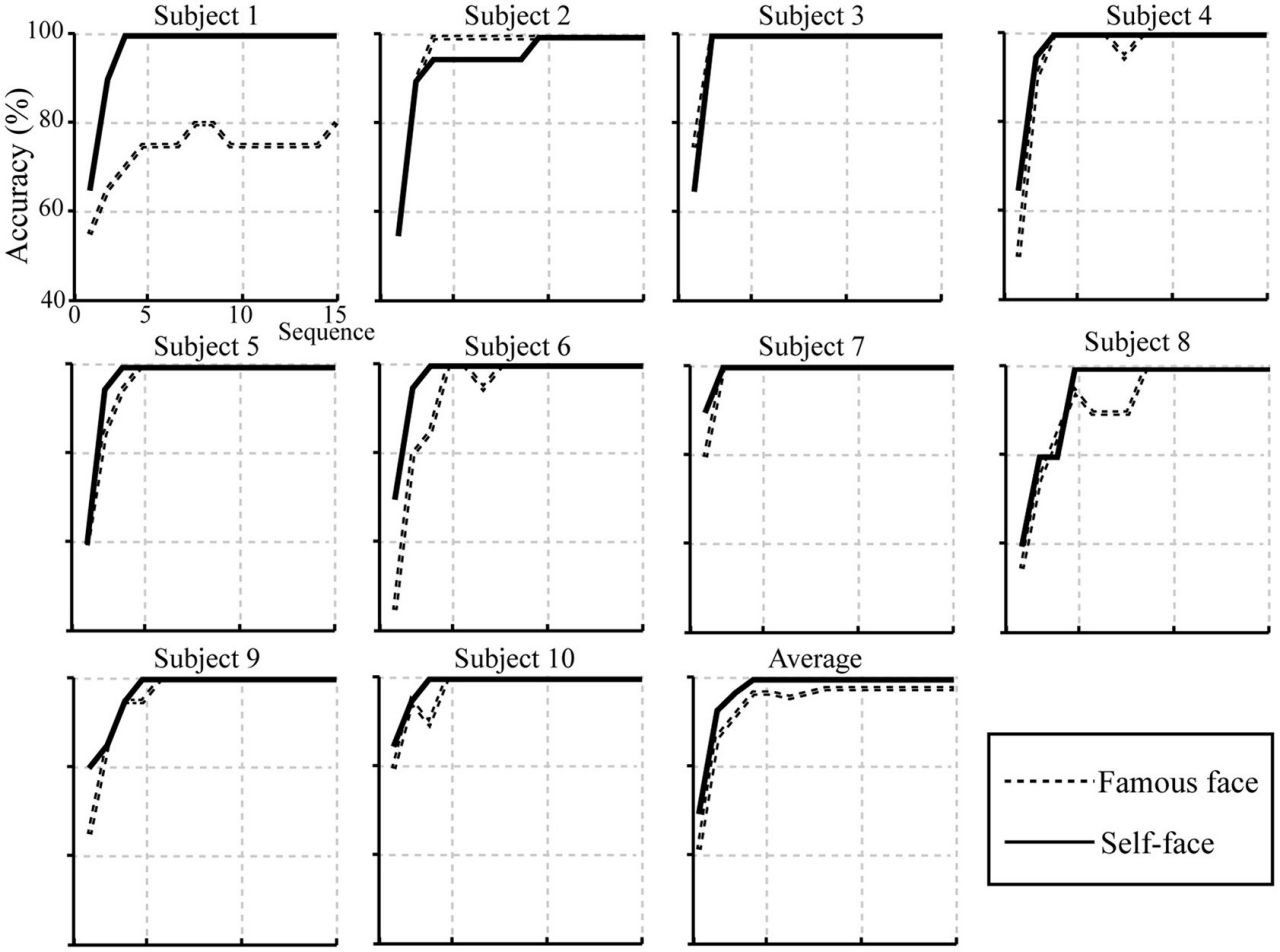
Individual and average accuracies of the self-face and famous face spelling paradigms for 10 subjects.

**Figure 10:**
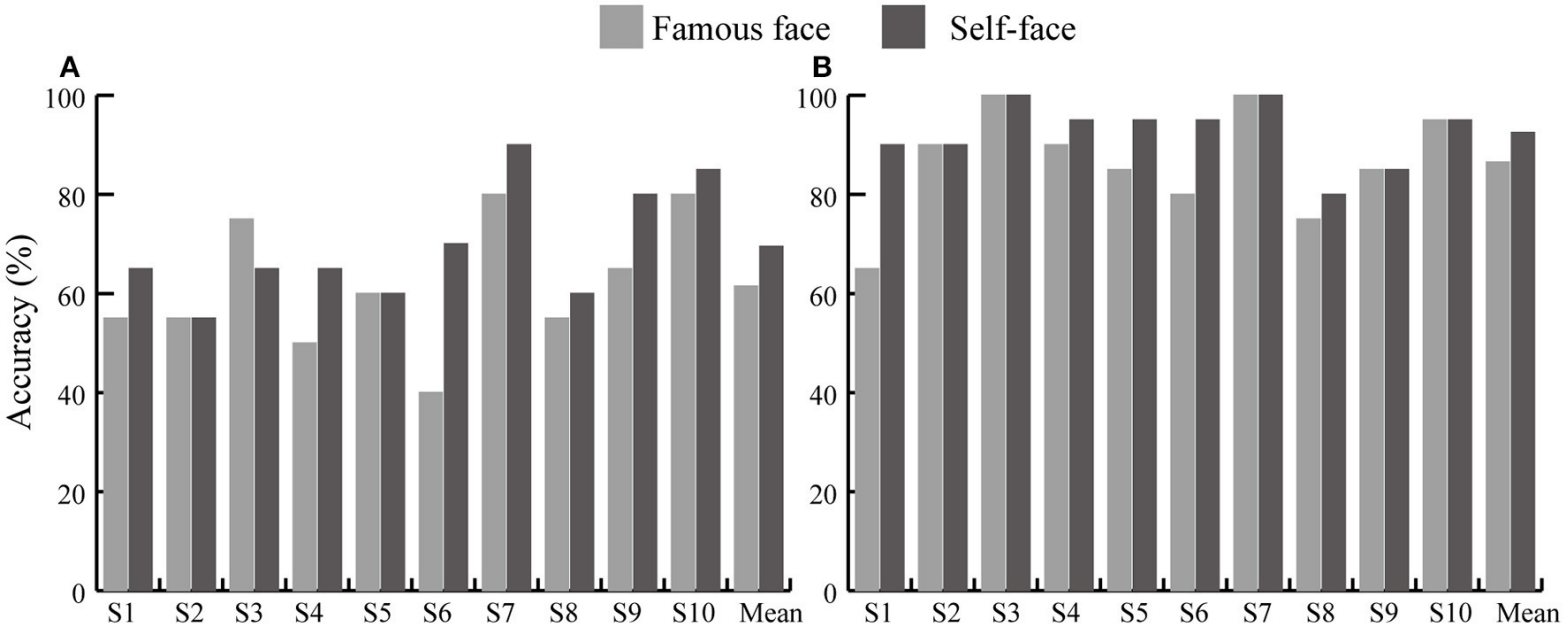
Accuracies of each subject and mean accuracy of 10 subjects at one superposition and two superpositions for the famous face and self-face spelling paradigms. **(A)** Accuracies at one superposition. **(B)** Accuracies at two superpositions.

Figure 9 shows the individual and average offline accuracies in the two face spelling paradigms based on the feature vector B and a 1–30 Hz frequency band filter. The accuracies increased with the increase in the number of superpositions in both paradigms; the average spelling accuracy of the self-face spelling paradigm was greater than that in the famous face spelling paradigm at 1–15 superpositions. The average number of superpositions when the accuracies reached 100% for all subjects was 2 in the self-face spelling paradigm; thus, we conducted a *t*-test on the accuracies only for the first two superpositions between the self-face and famous face paradigms. We found significant differences between the self-face and famous face spelling paradigms at both one superposition (*t* = −2.331, *P* < 0.05; Figure 10A) and two superpositions (*t* = −2.25, *P* < 0.05; Figure 10B).

Table 1 shows the ITRs for each subject and the averages in the self-face and famous face spelling paradigms. The best ITR result, 31.4 bits min^−1^ at one superposition, was found with the self-face spelling paradigm. The average ITR was greater at two superpositions than at one superposition. The paired *t*-tests showed that the ITR was significantly greater in the self-face paradigm than in the famous face paradigm at one superposition (*t* = −2.414, *P* = 0.039 < 0.05) and two superpositions (*t* = −2.345, *P* = 0.044 < 0.05).

**Table 1:** The information transfer rate of each subject for the famous face and self-face spelling paradigms at one and two superpositions.

	**One superposition**		**Two superpositions**	
**Subject**	**Famous face**	**Self-face**	**Famous face**	**Self-face**
Subject 1	14.0	18.3	13.3	22.8
Subject 2	14.0	14.0	22.8	22.8
Subject 3	23.1	18.3	27.5	27.5
Subject 4	12.0	18.3	22.8	25.2
Subject 5	16.1	16.1	20.7	25.2
Subject 6	8.4	20.6	18.7	25.2
Subject 7	25.7	31.4	27.5	27.5
Subject 8	14.0	16.1	16.8	18.7
Subject 9	18.3	25.7	20.7	20.7
Subject 10	25.7	28.4	25.2	25.2
Avg. ± SD	17.1 ± 5.9	20.7 ± 5.8	21.6 ± 4.6	24.1 ± 2.8
*p*-value	*t* = −2.414; *p* = 0.039		*t* = −2.345; *p* = 0.044	

*The unit of information transfer rate is bit/min*.

The online accuracies and ITRs of each subject for the famous face and self-face spelling paradigms are shown in Table 2. We found that the average accuracy and ITR in the self-face spelling paradigm were higher than those in the famous face spelling paradigm. Paired *t*-tests showed that there were significant differences in the accuracy and ITR between the two spelling paradigms (accuracy: *t* = −2.643, *P* < 0.05; ITR: *t* = −3.140, *P* < 0.05).

**Table 2:** The online accuracies and ITRs for all subjects in the famous face and self-face spelling paradigms.

	**Accuracies (%)**		**ITRs (bit/min)**	
**Subject**	**Famous face**	**Self-face**	**Famous face**	**Self-face**
Subject 1	96.7	100.0	31.9	33.6
Subject 2	80.0	93.3	22.8	29.8
Subject 3	60.0	66.7	14.3	17.0
Subject 4	70.0	76.7	18.3	21.3
Subject 5	73.3	83.3	19.8	24.4
Subject 6	80.0	73.3	22.8	19.8
Subject 7	86.7	93.3	26.1	29.8
Subject 8	90.0	96.7	27.9	31.9
Subject 9	83.0	90.0	24.3	27.9
Subject 10	80.0	80.0	22.8	22.8
Avg. ± SD	80.0 ± 10.4	85.3 ± 11.0	23.1 ± 5.0	25.8 ± 5.4
*p*-value	*t* = −2.643, *P* < 0.05		*t* = −3.140, *P* < 0.05	

## Discussion

In the present study, we proposed a new P300-speller using self-face stimulus and assessed the grand-average ERP waveforms elicited by target stimuli in the new and control spelling paradigms, analyzed the different ERP waveforms and the scalp topographies corresponding to significantly different waveforms elicited by the target minus non-target stimuli, and compared the classification accuracy and ITR of offline and online experiments between the self-face and famous face spelling paradigms.

### ERPs

Previous work has found that the performance of the P300-speller system could be improved by enhancing the difference between target trials and non-target trials (Jin et al., 2012). Therefore, we compared the waveforms (ERP_Target_ – ERP_Non−target_) elicited during the two face paradigms and found a significant difference between the two. The first significantly different waveform was from 340 to 480 ms over the fronto-central-parietal area (Figure 6), i.e., the P300. The P300 is not only associated with attention and cognitive processing (Polich, 2007) but also reflects the involvement of higher-order cognitive functions, including self-relevance (for one's own face, e.g., Ninomiya et al., 1998; Tanaka et al., 2006). Ninomiya et al. (1998) found that the P300 amplitude in response to one's own face was significantly larger than that in response to other stimuli. The authors, therefore, suggested that enhancement of the P300 in response to one's own face is not only due to an orienting response to a physically deviant stimulus but also due to the additional effect of relevance to the subject. Thus, the P300 can serve as an index of self-relevance, whereby higher self-relevance corresponds to a larger P300 amplitude (Kok, 2001). In Miyakoshi et al.'s study, the P300 amplitude elicited by the self-face stimulus was greater than that elicited by a famous face, and the P300 could distinguish the self-face from a famous face, and the authors, therefore, suggested that the P300 amplitude was sensitive to self-relevance (Miyakoshi et al., 2008). Therefore, the larger amplitude P300 in the self-face spelling paradigm than in the famous face spelling paradigm may be due to the higher self-relevance of the self-face than of the famous face for subjects.

The second significant difference in positive waveform was observed from 480 to 600 ms at the parietal-central area (Figure 6); this was similar to the P600f, which is related to processes involved in the recollection of faces (Eimer, 2000; Curran and Hancock, 2007). Some studies have suggested that perception of an individual's face may induce spontaneous activation of the characteristic and information associated with the individual (Bargh et al., 1996; Todorov and Uleman, 2002). The ERPs between 500 and 700 ms with a larger amplitude in response to a familiar face as compared to an unfamiliar face may indicate that the perception of the familiar face automatically generated more of one's personal traits or other episodic information than the perception of an unfamiliar face (Sui et al., 2006). Curran and Hancock (2007) also reported that a familiar face elicited a larger positive waveform between 500 and 700 ms (P600f) than did a stranger's face. Thus, we speculate that the larger P600f amplitude observed in the self-face spelling paradigm than in the famous face spelling paradigm indicates that the self-face induced more recollection, including characteristic or episodic information about the self than did the famous face.

The third significant difference in positive waveforms was from 700 to 800 ms at the fronto-central area (Figure 6). In ERP studies of face recognition, attending to the self-face induced a larger amplitude waveform between 600 and 800 ms at the prefronto-central area than did attending to a familiar face; it was speculated that this component was affected by the allocation of attentional resources in face recognition (Sui et al., 2006). Miyakoshi et al. (2008) found that the self-face was more likely to attract the attention of participants than a familiar face. In our study, the increased amplitude between 700 and 800 ms for the self-face than for the famous face paradigm may indicate that subjects paid more attention to their own faces.

In addition, our results showed that there was no significant difference in the N170 amplitude between the two spelling paradigms. This may be due to differences in experimental design (Keyes et al., 2010; Alonso-Prieto et al., 2015). Alonso-Prieto et al. (2015) reported that the sensitivity of the N170 to faces with different levels of familiarity is affected by the experimental settings, such as faces with different facial angles or faces with emotional information. For example, there was a difference in the N170 between a famous face and the self-face in studies of the influence of facial angle (Miyakoshi et al., 2008) and of emotional expression (Caharel et al., 2005), while Tacikowski et al. (2011) found no difference in the N170 amplitude between the self-face and a famous face when using frontal and neutral face images. In our study, the famous face and self-face comprised frontal and neutral images; thus, our results are consistent with those of Tacikowski et al. In addition, the type of familiarity of the face has also been found to affect the sensitivity of the N170 (Alonso-Prieto et al., 2015). For example, Sui et al. (2006) found that the N170 did not differ between self-faces and familiar faces (classmates), while Keyes et al. (2010) showed an increased N170 amplitude to the self-face relative to familiar faces (good friends). In the present study, the reason we found no difference in the N170 between the two paradigms may be that the difference in familiarity level between the famous face (Ming Yao) and the self-face may not have been enough to induce a statistically significant difference in N170 amplitude.

### Classification Accuracies and ITR

Offline classification results showed that the average accuracies of the self-face spelling paradigm were higher than those of the famous face spelling paradigm at all numbers of superpositions (Figure 9). A significant difference was found between the self-face and famous face spelling paradigm at one superposition (*P* < 0.05; Figure 10A) and at two superpositions (*P* < 0.05; Figure 10B). The offline accuracies demonstrated that use of the self-face improved the performance of the facial spelling paradigm because the self-face stimulus induced larger ERP components than did the famous face. In addition, the improvement and stability of spelling accuracy required stimuli to be repeated several times because of the low signal-to-noise ratios; however, increasing the number of repetitions may reduce the spelling speed. Thus, the ITR depended on both classification accuracy and speed character output, which is an important statistical metric for the performance of the P300-speller system. Our results indicated that the ITR of the self-face spelling paradigm was significantly greater than that of the famous face spelling paradigm at the first two superpositions (*P* < 0.05). The best result, 31.4 bits min^−1^ for subject 7, was obtained with the self-face spelling paradigm, in which subject 7 achieved 90% accuracy with one superposition only. Yet, the average ITR at two superpositions was larger than that at one superposition, and the standard deviation at one superposition was greater than that at two superpositions in both spelling paradigms (Table 1). This indicated that the spelling stability and performance is better at two superpositions. Therefore, in the online experiment, we set the trial to repeat only twice (that is, two superposition for 6 rows/columns) to acquire the accuracies and ITRs of character spelling. The online results showed that accuracy and ITR of the self-face spelling paradigm were significantly larger than those of the famous face spelling paradigm (Table 2). In summary, the proposed self-face spelling paradigm significantly improved the performance of the P300-speller system.

In addition, we compared the offline classification accuracies based on different feature vectors and frequency band passes. For feature vector A (25 × 12) and feature vector B (45 × 14), there was no significant difference at all superposition times, which indicates that the feature vector from amplitude difference between target and non-target stimuli can acquire classification results that are comparable to the feature vector in the 0–800 ms time window and at all channels (Figure 7). The classification results based on three frequency band passes showed that the best classification result was at 1–30 Hz at first three superpositions in both spelling paradigms (Figure 8), which indicated that a filter of 1–30 Hz could be a good choice for the classification accuracy of the P300-speller system.

### Future Work

The analysis of ERPs, classification accuracies, and ITRs between the two spelling paradigms showed that the self-face stimulus elicited significantly increased ERP amplitudes compared to the famous face stimulus and improved the spelling accuracy and ITR of the P300-speller system. Moreover, the use of self-face also avoided the copyright issues caused by using a famous face. Thus, the proposed self-face paradigm promotes practical applications of BCIs system. Some recent studies have shown that the brain responded more positively to a happy face and which could elicit increased ERP amplitudes, compared to a neutral face stimulus (Denefrio et al., 2017; Lu et al., 2019). In future work, we intend to use the subject's own happy face to investigate whether the self-face with happy emotion can further improve the performance and practicability of the P300-speller system.

## Conclusion

This study investigated whether the use of the self-face could improve the performance of the P300-speller system as compared to the use of a famous face. We found a significant improvement in classification accuracy and ITR for the self-face spelling paradigm at the first two superpositions, as compared to the famous face spelling paradigm, which may have a significant impact on increasing the speed and accuracy of spelling. Moreover, this has significance in practical BCI applications because the use of a famous face may involve copyright infringement problems.

## Data Availability Statement

All datasets generated for this study are included in the article/supplementary material.

## Ethics Statement

The studies involving human participants were reviewed and approved by the ethics committee of Changchun University of Science and Technology. The patients/participants provided their written informed consent to participate in this study. Written informed consent was obtained from the individual(s) for the publication of any potentially identifiable images or data included in this article.

## Author Contributions

QL and ZL designed the experiment, wrote the manuscript, and revised the manuscript. ZL and NG implemented the experiment and accomplished the data processing. ZL and JY analyzed the experimental results and revised the manuscript. All authors read and approved the final manuscript.

### Conflict of Interest

The authors declare that the research was conducted in the absence of any commercial or financial relationships that could be construed as a potential conflict of interest.
